# Effects of promoter leakage on dynamics of gene expression

**DOI:** 10.1186/s12918-015-0157-z

**Published:** 2015-03-21

**Authors:** Lifang Huang, Zhanjiang Yuan, Peijiang Liu, Tianshou Zhou

**Affiliations:** Guangdong Province Key Laboratory of Computational Science, School of Mathematics and Computational Science, Sun Yat-Sen University, Guangzhou, 510275 PR China; Institute of Computational Mathematics, Department of Mathematics, Hunan University of Science and Engineering, Youzhou, 425100 PR China

**Keywords:** Gene model, Promoter leakage, Probability distribution, Burst dynamics, Noise

## Abstract

**Background:**

Quantitative analysis of simple molecular networks is an important step forward understanding fundamental intracellular processes. As network motifs occurring recurrently in complex biological networks, gene auto-regulatory circuits have been extensively studied but gene expression dynamics remain to be fully understood, e.g., how promoter leakage affects expression noise is unclear.

**Results:**

In this work, we analyze a gene model with auto regulation, where the promoter is assumed to have one active state with highly efficient transcription and one inactive state with very lowly efficient transcription (termed as promoter leakage). We first derive the analytical distribution of gene product, and then analyze effects of promoter leakage on expression dynamics including bursting kinetics. Interestingly, we find that promoter leakage always reduces expression noise and that increasing the leakage rate tends to simplify phenotypes. In addition, higher leakage results in fewer bursts.

**Conclusions:**

Our results reveal the essential role of promoter leakage in controlling expression dynamics and further phenotype. Specifically, promoter leakage is a universal mechanism of reducing expression noise, controlling phenotypes in different environments and making the gene produce generate fewer bursts.

**Electronic supplementary material:**

The online version of this article (doi:10.1186/s12918-015-0157-z) contains supplementary material, which is available to authorized users.

## Background

Gene expression dynamics is a lasting issue in Systems Biology and has attracted extensive attention. While recent advances in experimental methods allow direct observations of gene expression levels in individual cells, there is considerable interest in theoretically understanding how different molecular mechanisms of gene expression influence variations in mRNA or protein levels across a population of cells. In fact, quantifying the contributions of different sources of noise using stochastic models of gene expression is an important step towards understanding fundamental cellular processes and cell-to-cell variability.

**Figure 1:**
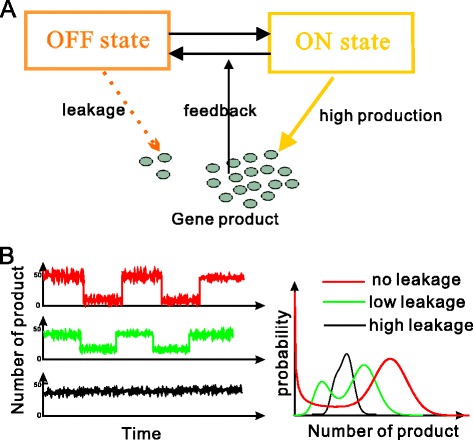
**A schematic diagram for effect of promoter leakage on expression dynamics. (A)** schematic description of two-state gene auto-regulatory model with leakage, where gene product is produced not only at the ON state with high efficiency but also at the OFF state with very low efficiency (termed as promoter leakage); **(B)** representative time series for changes in the number of gene products (left) and effect of leakage on population distribution (right).

Many theoretical models of gene expression have been proposed and become more and more subtle, from the initial one-state model [1,2] to the common two-state model [3] to those considering many detailed processes or factors, such as chromatin remodeling [4-6], TATA-box- mediated promoter [7-9], transcription additional re-initiation [10], growth rate [11], copy number variations [12], recruitment of transcription factors [13,14], alternative splicing [15]. In spite of these, few gene models in previous studies considered promoter leakage. Here, by promoter leakage we mean that transcription efficiency at the promoter inactive state is much lower than that at the promoter active state. In fact, it has been experimentally verified that transcription takes place not only at the active state but also at the inactive state of promoter, e.g., different nucleosome protein binding sites can lead to different expression efficiencies, some of which are high whereas the others are very low [16,17]; a basal transcription rate at each open promoter state implies that the promoter has leakage; and transcription can take place at some promoter state with a very low rate due to the pre-initiation complex formed at the TATA box [7]. Some studies have shown that increasing the leakage rate may eliminate bistability [18] whereas decreasing the leakage rate of protein production can lead to persistent oscillations [19,20]. But there has been no systematic study on how promoter leakage affects dynamics of gene expression including the noise in gene product, probability distribution, and bursting kinetics (characterized by burst size and burst frequency). This paper will address these issues by analyzing three cases: the common ON-OFF model, the ON-OFF model with negative regulation and the ON-OFF model with positive regulation, referring to schematic Figure 1(A). We will present a systematic investigation for each case.

To be clear, we will analyze relevant literatures and present the major findings of this study from three different aspects.

First, how the architecture of promoter affects expression noise is a hot issue and has attracted attention of many scholars [21,22]. Here, the promoter architecture mainly includes the pattern of transitions among promoter activity states and exits of transcription [22,23]. Some transcription rates may be very small (this corresponds to promoter leakage). On the other hand, it is known that feedback is a ubiquitous mechanism controlling signals. In general, the effect of feedback on expression noise depends on the type of feedback (positive or negative) and model parameters [24]. It has been shown that direct negative feedback decreases fluctuations and is a ubiquitous mechanism for homoeostatic control [25,26]. A question naturally arises: in the case of feedback loop, how does promoter leakage impact expression noise? By analyzing effects of promoter leakage on gene expression using the above-mentioned three gene models, we find that promoter leakage always reduces expression noise, regardless of models, feedback types and model parameters as well as whether or not the expression level is fixed. This implies an important fact that promoter leakage is a universal mechanism of reducing expression noise.

Second, it is well known that probability distribution not only provides the most complete information on stochastic dynamics of a stochastic model but also can clearly indicate the system’s states in the stochastic sense since a different peak in the distribution corresponds to a different state of the system. In addition, peakedness of probability distribution has biological implication, e.g., bimodal or multimodal gene expression (i.e., mRNA or protein distribution exhibits two or multiple peaks) is a cause of phenotypic diversity in genetically identical cell populations [27]; the amount of phenotypic variation in gene product population can determine fitness by affecting growth rate, robustness and adaptation [27-31]. Increased phenotypic variation can enhance adaptation and growth of cells in fluctuating environments as well as robustness of the population to external stresses [32-34]. It is particularly beneficial to microbial cells that need to adapt efficiently to sudden changes in environmental conditions [35,36]. And decreased phenotypic variation, where every gene product is as close as possible to an optimal level of gene expression that maximizes fitness, is usually advantageous to cells in constant environments [28,37,38]. Given the above facts or results, an unsolved question is how promoter leakage influences probability distribution and phenotypic heterogeneity. Although a theoretical study [39] illustrated the impact of promoter leakage on a probability distribution, the corresponding model did not consider feedback. We find that promoter leakage tends to unimodalize the mRNA or protein distribution regardless of feedback type, implying that promoter leakage may cause phenotypic simplification. In a word, phenotypic diversity is beneficial to survival of cells in a fluctuated environment on the one hand and phenotypic oneness is beneficial to fitness of cells in a constant environment on the other hand. Through our study, we conclude that promoter leakage is a mechanism of controlling phenotypes in different environments: cells would make use of not only the mechanism of promoter leakage-induced diverse phenotypes to adapt changes in environments but also the mechanism of promoter leakage-induced optimal phenotypes to adapt to constant environments.

Third, it has been shown that mRNAs or proteins are produced often in a bursty manner [40-42]. In fact, single molecule measurements have provided evidence for transcriptional or translational bursting [9,43,44]. An accepted view is that bursting dynamics are responsible for the generation of cellular heterogeneity in the response of genetically identical cells to the same stimulus, e.g., the authors in Ref. [34] demonstrated in yeast cells that high levels of cell-to-cell variability, originated from slow promoter state fluctuations, may confer cell colonies with an enhanced probability of survival when subjected to external stresses, such as addition of high concentrations of antibiotic. Regarding bursting dynamics, there have been many studies. For example, for a two-state gene model, it has been shown that the higher the burst frequency (BF) is, the lower is the gene expression noise whereas the larger the burst size (BS) is, the higher is the noise [42,45,46]. More interestingly, R.D. Dar, *et al.* [42] showed by analyzing 8,000 individual human genomic loci that transcriptional bursting dominates across the human genome, both BF and BS change by chromosomal location, and transcriptional activators alter BF and BS, depending on the expression level of the locus. In addition, G. Hornung, *et al.* [8] showed that burst size is a promoter-specific property that is relatively robust to sequence mutations but depends strongly on the interaction between the TATA box and promoter nucleosomes. In spite of these studies, it is not clear whether the results obtained in the case of neither feedback nor promoter leakage are still correct in the case of feedback or promoter leakage or both. We will address this issue. By model analysis, we find that a higher leakage rate of promoter produces fewer bursts, regardless of feedback type, referring to Figure 1(B).

## Results

### Gene model and analytical distribution

In principle, the chemical master equation (CME) provides the most complete model for probabilistic behavior of any biochemical reaction network including gene auto-regulatory circuits. Alternatively, the joint probability density function (or the joint probability distribution) for all the reactive species in a reaction network provides the most complete information on stochastic properties of this system. Therefore, finding probability distribution becomes a common interest in understanding all the possible stochastic properties of a biochemical reaction network. Here, we will derive the analytical expression for the steady-state distribution in a two-state gene auto regulatory model with promoter leakage.

Before presenting our analytical result, let us simply introduce our model to be investigated. Assume that a gene has two activity states (we will use *D* to represent promoter state): the active one (or the ON state) where DNAs are transcribed into mRNAs that are then translated into proteins (denoted by *P*), and the inactive one (or the OFF state) where transcription is not nonexistent but is lowly efficient, that is, there is a smaller transcription rate at the inactive state in contrast to that at the active state. The latter case is often called promoter leakage [47,48]. The promoter leakage was neglected in previous studies of gene models [49-52], or equally, the transcription rate at the inactive state was assumed as zero. In addition, we assume that the gene product regulates its expression as a transcription factor, thus forming a feedback loop. This regulation may be positive or negative. In particular, we assume that the transcription factor regulates the gene expression not in a manner of sequestration (by sequestration we mean that dissociation of protein from transcription factor-DNA complex is a slow process, or binding of transcription factor-DNA is strong) [53,54] but in a manner of directly changing transition rates between promoter states (i.e., assuming that association and dissociation of a transcription factor are a very fast process. Specifically, the transcription factor first binds fast to DNA and then dissociate rapidly from the DNA, leading that the transcription factor changes directly the switching rates between promoter states without consuming itself) [55,56]. To simplify our analysis, we further integrate transcription and translation into a single-step process. This simplification has been extensively made [52,57]. In fact, it has been shown that protein’s half-life is in general much longer than that of mRNA’s half-life [52,57], e.g., Shahrezaei and Swain [52] did a survey for ~ 2000 genes in budding yeast and found that the expressions of most of these genes satisfy this condition. A main reason for this consideration is to derive the analytical expression for the probability distribution of gene product or to give the analytical formula for calculating the noise intensity, which in turn can clearly describe our qualitative results. In the Additional file 1, we also investigate slightly more complex gene models, which consider either a slow process for binding and dissociation of a transcription factor or two-step process of transcription and translation. By stochastic simulation, we find that the results using the complex models are basically similar to those using the simplified model, indicating that the latter can well capture the effect of promoter leakage on expression dynamics.

A transcription factor may regulate gene expression in an enhancing or a repressing manner. To derive our analytical distributions in a unified framework, we introduce the following set of biochemical reactions based on the above hypotheses.

1 $$ \begin{array}{l}{D}_0\overset{\gamma_1}{\to }{D}_1,{D}_1\overset{\gamma_0}{\to }{D}_0,{D}_1+P\overset{f}{\to }{D}_0+P\\ {}{D}_1\overset{\lambda_1}{\to }{D}_1+P,{D}_0\overset{\lambda_0}{\to }{D}_0+P,P\overset{d}{\to}\phi \end{array} $$

which describe our gene model with positive or negative regulation. Here, *D*_1_ and *D*_0_ represent the ON and the OFF states respectively if *λ*_1_ ≫ *λ*_0_ and the OFF and the ON states respectively if *λ*_0_ ≫ *λ*_1_. In the former case, the third reaction in the first row of Eq. (1) describes the negative regulation and *λ*_1_, *λ*_0_ are transcription rate and promoter leakage rate respectively, whereas in the latter case, this reaction describes the positive regulation and *λ*_0_, *λ*_1_ are transcription rate and promoter leakage rate respectively. Parameters *γ*_1_ and *γ*_0_ are transition rates between the promoter activity states, *f* represents feedback strength, and *d* is the degradation rate of gene product.

Then, we establish our mathematical model to be studied. Let *P*_0_(*n*, *t*) and *P*_1_(*n*, *t*) represent the probability that the gen product has *n* molecules at time *t* when the gene is at *D*_0_ and *D*_1_ states, respectively. Then, the discrete CME for the full reaction network takes the form

2 $$ \frac{\partial }{\partial t}\left(\begin{array}{c}\hfill {P}_0\left(n,t\right)\hfill \\ {}\hfill {P}_1\left(n,t\right)\hfill \end{array}\right)=\left(\begin{array}{cc}\hfill -{\gamma}_1\hfill & \hfill {\gamma}_0+fn\hfill \\ {}\hfill {\gamma}_1\hfill & \hfill -{\gamma}_0-fn\hfill \end{array}\right)\left(\begin{array}{c}\hfill {P}_0\left(n,t\right)\hfill \\ {}\hfill {P}_1\left(n,t\right)\hfill \end{array}\right)+\left(\begin{array}{c}\hfill \left[{\lambda}_0\left({E}^{-1}-I\right)+d\left(E-I\right)\right]{P}_0\left(n,t\right)\hfill \\ {}\hfill \left[{\lambda}_1\left({E}^{-1}-I\right)+d\left(E-I\right)\right]{P}_1\left(n,t\right)\hfill \end{array}\right) $$

where *E* is the step operator and *I* is the identity operator.

Next, we focus on finding the steady-state solution of Eq. (2). The basic idea is first to introduce probability-generating functions, and then to solve a coupled set of ordinary differential equations with respect to these functions. The overall procedure for finding the stationary distribution is technical. Here, we list results only, and the details for derivation are put in the Additional file 2 of this paper.

For convenience, let all the parameters be normalized by the degradation rate *d*, that is, *γ*_1_/*d* → *γ*_1_, *γ*_0_/*d* → *γ*_0_, *f*/*d* → *f*, *λ*_0_/*d* → *λ*_0_, *λ*_1_/*d* → *λ*_1_. Then, then analytical probability distribution can be expressed as

3 $$ P(n)=\frac{gA}{n!}{\displaystyle \sum_{m=0}^n\left(\begin{array}{c}\hfill n\hfill \\ {}\hfill m\hfill \end{array}\right){\lambda}_0^{n-m}}{\left[\left(f+1\right)Q\right]}^m\frac{{\left(\alpha -1\right)}_m}{{\left(\beta -1\right)}_m}{}_1F_1\left(\alpha +m-1,\beta +m-1;-Q\right) $$

which is the linear superposition of confluent hypergeometric functions. In Eq. (3), *λ* = *λ*_1_ − *λ*_0_*R* = *λ* − *f λ*_0_, $$ Q=\frac{\lambda -f\kern0.1em {\lambda}_0}{{\left(f+1\right)}^2} $$, $$ g=\frac{\lambda +{\gamma}_1+{\gamma}_0}{\gamma_1}-\frac{R}{\gamma_1\left(f+1\right)} $$, $$ \beta =1+\frac{\lambda +{\gamma}_0+{\gamma}_1}{f+1}-\frac{R}{{\left(f+1\right)}^2} $$$$ \alpha =1+\frac{\lambda {\gamma}_1}{R} $$ and $$ A={e}^{-{\lambda}_0}{\left[g{}_1F_1\left(\alpha -1,\beta -1;f\kern0.1em Q\right)\right]}^{-1} $$. The symbol $$ \left(\begin{array}{c}\hfill n\hfill \\ {}\hfill m\hfill \end{array}\right) $$ represents the combinational number of choosing *m* molecule from *n* molecules, and (*c*)_*n*_ is the Pochhammer symbol and is defined as (*c*)_*n*_ = *Γ*(*c* + *n*)/*Γ*(*c*). In principle, this analytical distribution gives all the stochastic information about the underlying gene model.

The more useful is that we can give the analytical formula for calculating the noise intensity for the gene product, where by noise intensity we mean that it is the ratio of variance over the square of mean. In fact, note that mean and variance can be calculated according to the following general formulae.

4 $$ \left\langle n\right\rangle ={G}^{\prime }(1),{\sigma}_n^2={G}^{{\prime\prime} }(1)+{G}^{\prime }(1)-{\left[{G}^{\prime }(1)\right]}^2 $$

where *G*(*z*) = *G*_0_(*z*) + *G*_1_(*z*) is the total generating function. Two factorial generating functions *G*_0_ (*z*) and *G*_1_(*z*) are analytically given in the Additional file 2. By calculation, we find

5 $$ {G}^{\prime }(1)=A{e}^{\lambda_0}\left[g{\lambda}_0{}_1F_1\left(\alpha -1,\kern0.5em \beta -1,\kern0.5em f\kern0.1em Q\right)+\lambda {}_1F_1\left(\alpha, \kern0.5em \beta, \kern0.5em f\kern0.1em Q\right)\right] $$

6 $$ \begin{array}{l}{G}^{{\prime\prime} }(1)=A{e}^{\lambda_0}\left[g{\lambda}_0^2{}_1F_1\left(\alpha -1,\beta -1,f\kern0.1em Q\right)+2\lambda {\lambda}_0{}_1F_1\left(\alpha, \beta, f\kern0.1em Q\right)\right.\\ {}\begin{array}{cc}\hfill \hfill & \hfill \hfill \end{array}+\frac{\lambda \left(\lambda {\gamma}_1+R\right)}{\lambda +{\gamma}_1+{\gamma}_0+f+1-R/\left(1+f\right)}{}_1F_1\left(\alpha +1,\beta +1,f\kern0.1em Q\right)\Big]\end{array} $$

Thus, the noise intensity is given according to the formula.

7 $$ {\eta}_n^2=\frac{{\sigma_n}^2}{\left\langle n\right\rangle {}^2}=\frac{G^{{\prime\prime} }(1)+{G}^{\prime }(1)-{\left[{G}^{\prime }(1)\right]}^2}{{\left[{G}^{\prime }(1)\right]}^2} $$

Next, we focus on effects of promoter leakage on expression noise as well as burst dynamics. Note that the promoter leakage rate is *λ*_0_ in the case of negative feedback whereas it is *λ*_1_ in the case of positive feedback. As pointed out in the previous section, by promoter leakage we mean that the parameter *λ*_0_ is in general more than zero but much smaller than *λ*_1_ in the former case. Conversely, *λ*_1_ is in general more than zero but much smaller than *λ*_0_ in the latter case. We point out that to demonstrate the more remarkable numerical effects of promoter leakage, we sometimes let the leakage rate be a little smaller but be not much smaller than the normal transcription rate. For convenience, we always set *d* = 1 in our simulation.

### Leakage always attenuates expression noise

**Figure 2:**
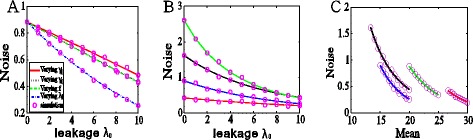
**Effects of promoter leakage on gene expression noise. (A)** The dependence of the noise intensity on the promoter leakage rate in the case that the gene product amount is fixed at a certain value, showing the noise intensity is always a monotonically decreasing function of the leakage rate, regardless of ways to keep the average expression level fixed (e.g., decreasing the transition rate from OFF to ON (*γ*_1_) (solid red line); increasing the transition rate from ON to OFF (*γ*_0_) (black dotted line); increasing the feedback strength (*f*) (green dashed line); decreasing the maximum transcription rate (*λ*_1_) (blue dash dot line). **(B)** Results in the case that the mean expression is not fixed, showing that increasing the leakage rate reduces the expression noise, where 4 colored lines correspond to 4 different sets of parameter values: *λ*_1_ = 40, *γ*_0_ = 0.1, *γ*_1_ = 0.1, f = 0.01(green); *λ*_1_ = 40, *γ*_0_ = 0.1, *γ*_1_ = 0.2, f = 0(red); *λ*_1_ = 40, *γ*_0_ = 0.2, *γ*_1_ = 0.1, f = 0(black); *λ*_1_ = 30, *γ*_0_ = 0.1, *γ*_1_ = 0.1, f = 0(blue). This subfigure shows that the conclusion that promoter leakage always reduces noise is independent of model parameters. **(C)** The noise as a function of the mean for different values of the leakage rate, where the parameter values are the same as those used in Figure 2 **(B)**. The subfigure shows that the larger the promoter leakage rate is, the more is the number of gene product molecules, implying that promoter leakage always reduces expression noise. In Figure 2 **(B)** and **(C)**, lines represent theoretical results whereas circles represent stochastically simulating results.

In previous studies [49-52], the effect of promoter leakage on gene expression was frequently neglected. Here, we numerically show that the promoter leakage has unneglectable effects on gene expression and in particular on expression noise. More precisely, the promoter leakage always reduces the noise in gene product. The numerical results are shown in Figure 2.

In our numerical calculation, we let the leakage rate increase but keep the mean expression of gene product fixed. This constraint implies that two of the system parameters are dependent of each other if the other parameters are fixed. For example, consider the case of *λ*_1_ ≫ *λ*_0_. If gene product is kept at a fixed level, then increasing leakage rate (*λ*_0_) implies: 1), a decrease in the transition rate from OFF to ON (*γ*_1_); 2), an increase in the transition rate from ON to OFF (*γ*_0_); 3), an increase in the feedback strength (*f*); or 4), a decrease in the maximum transcription rate *λ*_1_. Specifically, in Figure 2(A), changing *λ*_0_ implies varying *γ*_1_ (solid red line) if we fix *λ*_1_ = 40, *γ*_0_ = 0.1, and *f* = 0; changing *λ*_0_ implies varying *γ*_0_ (black dotted line) if we fix *λ*_1_ = 40, *f* = 0, and *γ*_1_ = 0.1; changing *λ*_0_ implies varying *f* (green dashed line) if we fix *λ*_1_ = 40, *γ*_0_ = 0.1 and *γ*_1_ = 0.1; changing *λ*_0_ implies varying *λ*_1_ (blue dash dot line) if we fix *γ*_0_ = 0.1, *γ*_1_ = 0.1 and *f* = 0. We point out that this classification of parameter values implies that positive and negative feedbacks can be considered in a unified framework. Therefore, it is unnecessary to distinguish the case of positive feedback from that of negative feedback for simulation.

From Figure 2(A), we observe that in the case that the average expression level is fixed, the noise intensity *η*_*n*_ is a monotonically decreasing function of the promoter leakage rate, regardless of ways that the mean is fixed. In other words, the noise intensity always decreases with the increase of the leakage rate. This implies that the promoter leakage plays a role of attenuating the noise in the gene product, no matter what the property of feedback (positive or negative). Figure 2(B) is used to show that the conclusion that the promoter leakage always reduces the expression noise does not depend on the choice of parameter values, thus being qualitatively invariant. In this figure, we choose 4 different sets of parameter values (see the caption of Figure 2(B)) to demonstrate numerical results. The combination of Figures 2(A) and (B) implies that promoter leakage is a mechanism of efficiently reducing expression noise. To check if this mechanism is universal, we also investigate slightly more complex models in the Additional file 1, which consider either strong binding of transcription factors to DNAs or the two-step processes of transcription and translation. By numerical simulation, we find that the qualitative conclusion that promoter leakage always reduces noise still keeps invariant, referring to Additional file 1: Figure S1 (C), Figure S2 (C) and Figure S3 (C).

Intuitively, the larger the promoter leakage rate is, the more is the number of gene-product molecules. This will lead to the reduction of the noise in gene product (see Figure 2(C)). This result is easily seen in the case that the mean level is not fixed but seems not apparent in the case that the mean level is fixed. However, if the mean expression level is fixed, then the above qualitative conclusion seems unrelated to the property or type of feedback. See the following content for interpretation.

### Promoter leakage tends to unimodalize distribution

Several examples have shown that the noise in gene expression is a potential mechanism to generate phenotypic heterogeneity [27,49,58]. The phenotypic diversity has been a focus of attention in biology, since the amount of phenotypic variation (also known as gene expression noise) in a cell population can determine fitness by affecting growth rate, robustness and adaptation [28-31]. Population diversity offers an alternate way that cells adapt to randomly fluctuating environments [30]. Increasing phenotypic variation is particularly beneficial to organisms that need to adapt efficiently to sudden changes in chemical composition, local temperature, or illumination [30]. In contrast, decreasing phenotypic variation is usually advantageous in constant environments [28,37,38]. The peaks of gene product distribution are a cause of generating phenotypic diversity in genetically identical cell populations [27], and the gene product noise always decreases with the increase of the leakage rate as shown above, so we naturally consider the relationship between promoter leakage and phenotypic selection. For this, we will investigate the effect of promoter leakage on peakedness of probability distribution.

**Figure 3:**
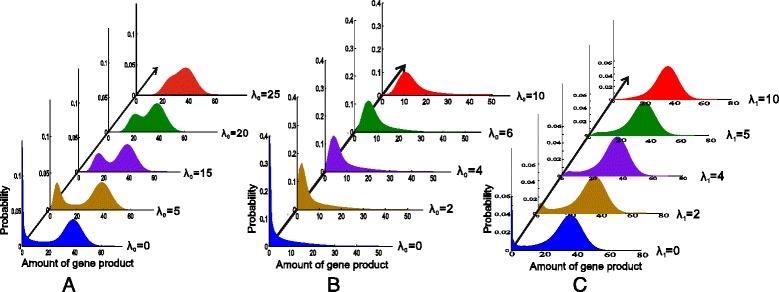
**Effects of promoter leakage on distribution. (A)** no feedback (*f* = 0): the gene product distribution changes from bimodality to unimodality when the leakage rate increases. The parameter values are set as *λ*_1_ = 40, *γ*_0_ = 0.1, *γ*_1_ = 0.2; **(B)** negative feedback: only one peak closed to the origin gradually becomes another peak away from the origin with the increase of the leakage rate. Other parameter values are set as *λ*_1_ = 40, *γ*_0_ = 0.1, *γ*_1_ = 0.2, f = 0.1; **(C)** positive feedback: two peaks gradually become one peak away from the origin with the increase of the leakage rate, where the parameter values are set as *λ*_0_ = 40, *γ*_0_ = 0.1, *γ*_1_ = 0.5, f = 0.1.

It has been shown that a two-state gene model can exhibit bimodal distributions if neither promoter leakage nor auto regulation is considered [28,59]. Bimodality can also occur even in the presence of regulation [60,61]. If promoter leakage is considered, however, we find that the situation is different. The numerical results are demonstrated in Figure 3, where we consider three cases: no feedback, which corresponds to *f* = 0; negative feedback, which implies that *D*_1_ represents the active state but *λ*_1_ corresponds to the normal transcription rate whereas *λ*_0_ to the leakage rate; and positive feedback, which implies that *D*_0_ represents the active state but *λ*_0_ corresponds to the normal transcription rate whereas *λ*_1_ to the leakage rate.

From Figure 3(A) where no feedback is considered, we observe that with the increase of the leakage rate from zero to 25, the gene product distribution finally becomes a single peak from initial two peaks. Figure 3(B) shows that in the case of negative feedback, the gene product distribution becomes a single peak away from the origin from one single peak near the origin with the increase of the leakage rate from zero to 10. In contrast, Figure 3(C) shows that in the case of positive feedback, the gene product distribution becomes a single peak away from the origin from two distinct peaks with the increase of the leakage rate from zero to 10. Compared to the case in Figure 3(A), this change in Figure 3(C) is faster.

Thus, we obtain an interesting conclusion, that is, promoter leakage tends to unimodalize the distribution, independently of the property or type of feedback. We point out that neither transcription factor long residency on the DNA nor transcription and translation in different time scales influences this qualitative conclusion. In fact, we have shown in the Additional file 1 that if these factors are considered, then the unimodal distribution of gene product will become more apparent with the increase of the promoter leakage rate, referring to Additional file 1: Figure S1 (B), Figure S2 (B) and Figure S3 (B). Since multimodality is an important source resulting in the diversity of phenotype and since the unimodal distribution implies the singleness of phenotype. While increased phenotypic diversity can enhance adaptation and growth of cells in fluctuating environments [33], so our conclusion provides an important hint, that is, promoter leakage would not be too remarkable in *in vivo* organisms.

As mentioned in the introduction, diversity of phenotype is beneficial to survival of cells in a fluctuated environment whereas oneness of phenotype is beneficial to fitness of cells in a constant environment [35-38]. Thus, the above results imply that promoter leakage would be a mechanism of effectively controlling phenotype in different environments: cells would make use of not only the mechanism of promoter leakage-induced diverse phenotypes to adapt fluctuated environments but also the mechanism of promoter leakage-induced optimal phenotypes to adapt constant environments.

### Promoter leakage can result in fewer bursts

As is well known, mRNAs or proteins are synthesized often in a burst manner [40-42]. Bursting kinetics is commonly characterized by two indices: burst size (BS) and burst frequency (BF). A question is how promoter leakage impacts bursting kinetics. Previous studies did not give a positive answer to this issue although it has been shown that the larger the BS is, the higher is the gene expression noise whereas the higher the BF is, the lower is the noise [42,46,47]. Here, we will show that promoter leakage has unneglectable influences on bursting kinetics, remarkably making the gene product produce fewer bursts.

**Figure 4:**
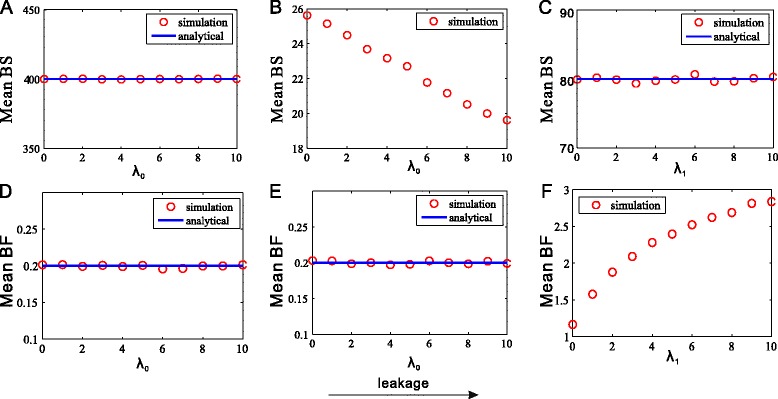
**Effects of promoter leakage on bursting kinetics. (A and D)** no feedback: promoter leakage does not change burst size nor burst frequency, where the parameter values are the same as those used in Figure 3 **(A)**. **(B and E)** negative feedback: promoter leakage does not change burst frequency, but reduces burst size, where the parameter values are the same as those used in Figure 3(B). **(C and F)** positive feedback: promoter leakage does not change burst size but increases burst frequency, where the parameter values are the same as those used in Figure 3(C).

**Figure 5:**
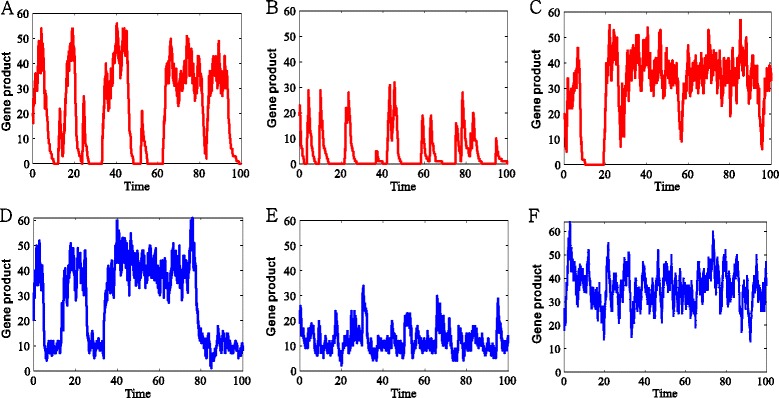
**Effects of promoter leakage on the time-dependent number of gene product molecules, where the leakage rate is set as either** ***λ***_0_ **= 0 (A, B) or** ***λ***_0_ **= 10 (D, E).** **(A and D)** no feedback, where the parameter values are the same as those used in Figure 3(A); **(B and E)** negative feedback, where the parameter values are the same as those used in Figure 3(B); **(C and F)** positive feedback, where the leakage rate *λ*_1_ is set as 10 for **(F)** or as 0 for **(C)**, and the other parameter values are the same as those used in Figure 3(C).

Before presenting results, let us simply introduce computation formulae associated with bursting kinetics. Recall that in the case of no feedback, the mean BF and the mean BS are calculated according to the following formulae [42]

8 $$ \left\langle BF\right\rangle =\frac{1}{\tau_{OFF}},\left\langle BS\right\rangle ={k}_{transcription}\cdot {\tau}_{ON} $$

where *τ*_*OFF*_ and *τ*_*ON*_ represent the mean time dwelling at OFF and ON states respectively, and *k*_*transcription*_ is the transcription rate when the gene is at ON state. First, consider that our model has no feedback, i.e., *f* = 0. If *λ*_0_ compared to *λ*_1_ is so small that it may be ignored (in this case, *D*_1_ represents the ON state), then we have *τ*_*OFF*_ = 1/*γ*_1_ [62,63], which implies 〈*BF*〉 = *γ*_1_, and *τ*_*ON*_ = 1/*γ*_0_ [62,63], which implies 〈*BS*〉 = *λ*_1_/*γ*_0_. Similarly, if *λ*_1_ compared to *λ*_0_ is so small that it may be ignored (in this case, *D*_0_ represents the ON state), then we have *τ*_*OFF*_ = 1/*γ*_0_, which implies 〈*BF*〉 = *γ*_0_, and *τ*_*ON*_ = 1/*γ*_1_, which implies 〈*BS*〉 = *λ*_0_/*γ*_1_. Thus, for the former case, we see from Eq. (11) that increasing the leakage rate *λ*_0_ does not change the mean BF nor change the mean BS, referring to Figure 4(A) and (D), but may make the original low expression amount of the gene product have a rise. Moreover, it can be seen from the time series shown in Figure 5 (more precisely, by comparing Figure 5(A) which corresponds to the case of no leakage with Figure 5(D) which corresponds to the case that the leakage rate is 10) that the number of gene product molecules tends to centralize a certain value. The similar phenomena can take place in the latter case.

Next, consider the case that there is feedback. In this case, increasing the promoter leakage rate will change the number of gene product molecules, thus in turn influencing the switching rates between the ON and the OFF states. Furthermore, this will change the dwelling times at two states of the promoter, thus influencing BS and BF. More specifically, in the case of negative feedback, increasing the leakage rate can increase the number of gene product molecules, leading that the effect of negative feedback becomes more remarkable. In other words, increasing the leakage rate will make the transition rate from the ON state to the OFF state become larger, i.e., will make the dwelling time at the ON state become shorter. This will lead to the decrease of BS, referring to Figure 4(B), and further the reduction of the expression noise. In addition, negative feedback does not change the transition rate from the OFF state to the ON state, so does not influence the dwelling time at the OFF state nor change the mean BF, referring to Figure 4(E). Figure 5(B) and (E) further justify the numerical results shown in Figure 4(B) and (E). Similarly, in the case of positive feedback, increasing the promoter leakage rate will increase the number of the gene product molecules, leading that the role of positive feedback becomes more remarkable. Specifically, increasing the leakage rate will make the transition rate from the OFF state to the ON state become larger, i.e., make the dwelling time at the OFF state become smaller. This will lead to the increase of BF, referring to Figure 4(F), and further the reduction of the expression noise. In addition, positive feedback does not change the transition rate from the ON state to the OFF state, so does not influence the dwelling time at the ON state nor change the mean BS, referring to Figure 4(C). The time series shown in Figure 5(C) and (F) further justify the numerical results shown in Figure 4(C) and (F).

Then, we want to know how increasing leakage influences bursting kinetics in the case of keeping the mean level fixed. By numerical analysis, we find that for negative feedback (*λ*_1_ ≫ *λ*_0_), the mean BS always decreases with the increase of the leakage rate *λ*_0_, regardless of the way that the mean is fixed, but the mean BF decreases only when the transition rate from OFF to ON (*γ*_1_) decreases, and is kept invariant in the other three cases: increasing the transition rate from ON to OFF (*γ*_0_); increasing the feedback strength (*f*); and decreasing the maximum transcription rate (*λ*_1_), referring to Additional file 1: Figure S4 (A). In the case of positive feedback (*λ*_1_ ≪ *λ*_0_)), if increasing the leakage rate (*λ*_1_) corresponds to the change of the transition rate from OFF to ON (*γ*_0_) or that of positive feedback intensity (*f*), then the mean BS does not change but the mean BF decreases; and if increasing the leakage rate (*λ*_1_) corresponds to change of the transition rate from ON to OFF (*γ*_1_) or the maximum transcription rate (*λ*_0_), then the mean BS decreases and the mean BF increases. referring to Additional file 1: Figure S4 (B).

In a word, under the condition that the mean level is fixed, increasing the leakage rate can make the mean BS decrease in most cases and the mean BS invariant in few cases, regardless of the way the mean is fixed and the type of feedback. This implies that increasing promoter leakage tends to generate fewer bursts.

Summarizing the above analysis, we know that in the case of no feedback, promoter leakage does not influence promoter dynamics, but in the presence of feedback, the former influences the latter and this influence is through the way that feedback indirectly impacts bursting kinetics. Specifically, the promoter leakage makes the burst size be reduced and the burst frequency be enlarged, indicating that the promoter leakage makes the gene produce fewer bursts. This conclusion can be also seen from the time series shown in Figure 5. In fact, by comparing Figure 4(D), (E) and (F) with their corresponding Figure 5(D), (E) and (F), we find that fewer bursts are generated due to the effect of promoter leakage.

## Discussion

Biochemical reactions associated with gene expression are all essentially single-molecule events and thus stochastic, resulting in substantial randomness in the production of mRNA or protein. This noise can significantly influence the expression levels of gene products and has been identified as a source of cell-to-cell variability. To capture effects of expression noise, many gene models have been proposed, such as those with simple promoter structures [2,7] or with a DNA loop [51] or with a more complex promoter structure [23], and those with auto regulation [7]. Almost these models, however, neglected the effect of promoter leakage on gene expression. Here, we have introduced and analyzed a stochastic model of gene expression, which considers not only promoter activity and regulation but also promoter leakage. Interestingly, we have derived the analytical distribution (seeing Eq. (2)), which can reproduce some known distributions obtained in simplified cases, e.g., with or without auto-regulation, without promoter leakage. More importantly, our results on the noise in gene product indicate that promoter leakage can be taken as a mechanism of attenuating expression noise, implying that previous gene models of no promoter leakage would overestimate expression noise. Similarly, previous estimations on bursting dynamics characterized by burst frequency and burst size would be inaccurate since they did not consider the effect of promoter leakage. In fact, we have shown that promoter leakage can significantly impact bursting dynamics (Figure 4) and expression noise (Figure 2). An intuitive interpretation for this impact is as follows. Promoter leakage can increase the amount of gene product, thus possibly reducing the intrinsic noise of gene product. Meanwhile, it also can influence promoter noise when the gene product as a transcription factor auto-regulates the transition rates between promoter states.

Regarding our model, here we present simple discussions. To simplify our analysis, our first assumption is that the binding and dissociation of a transcription factor is a very fast process, that is, it binds quickly to DNAs and dissociates rapidly from the DNAs. With this assumption, one can view that the transcription factor changes only the transition rates between promoter activity states without consuming itself. The similar assumption has been before made to obtain analytical distributions in gene models [55,56]. On the other hand, experimental studies indicated that the association and disassociation of transcription factors to promoter sites may be a slow process. Moreover, it was theoretically shown that the mode of binding of transcription factors to DNAs can affect the properties of expression noise [64,65]. If a slow process for binding of a transcription factor is introduced to the models studied here and even if transcription and translation processes are considered, then two qualitative conclusions obtained here, that is, increasing the leakage rate reduces noise and makes the gene product distribution be uni-modalized, will still held. For this, we have performed numerical simulation, with results shown in Additional file 1: Figure S1, Figure S2 and Figure S3. These results indicates that our simplified model have well captured effects of promoter leakage on expression dynamics including distribution, noise and bursting dynamics.

Our second assumption is that the gene promoter has one active state and one inactive state. In many cases, however, the promoter may have multiple activity states [23,63]. For example, the PRM promoter of phage lambda in *E. coli* is regulated by two different TFs binding to two sets of three operators that are brought together by looping out the intervening DNA. As a result, the number of regulatory states of the PRM promoter is up to 128 [66]. In particular, eukaryotic promoter structures would be more complex since they involve nucleosomes competing with or being removed by transcription factors [67]. In spite of this, our qualitative conclusions will not be ruined although the quantitative results would be modified if a more complex promoter structure is considered (data are not shown).

It should be pointed out that gene expression is a complex biochemical process. Except for the factors considered here such as transition between two activity state of the promoter, auto-regulation and promoter leakage, gene expression also involves other factors such as RNA nuclear retention, chromatin remodeling [4-6], combinatorial regulation from many transcription factors [68,69], DNA loop [70,71] and alternative splicing [15] as well as binding of transcription factors [64,72]. However, it is unclear how these factors impact gene expression in the combined case of feedback and promoter leakage.

Finally, our study would have biological implications in fields such as synthetic biology. First, according to our results obtained here, promoter leakage in circuits of stochastic gene expression especially in those with repressive regulation should be controlled and otherwise it would influence functions of the corresponding circuits, e.g., bistability in circuits of positive feedbacks [73], and information processing in circuits with AND gate [74]. See the review article [75] for more details. Thus, introducing promoter leakage to gene circuits to be designed would achieve better design effects. Second, our results can provide a guideline for biologists who design synthesized circuits used to probe for the relationship between bistability and phenotype. Third, our results would imply that promoter leakage is a mechanism of efficiently adjusting phenotypic diversity of *in vivo* organisms.

## Conclusions

Promoter leakage is an unneglectable factor in gene expression and plays a significant role of controlling expression dynamics and phenotypic diversity. Specifically, promoter leakage (1) always reduces expression noise; (2) tends to unimodalize the gene-product distribution; (2) makes the gene produce fewer burst in contrast to the case of no promoter leakage. These results imply that promoter leakage may be taken as a strategy of efficiently controlling cell-to-cell variability.

## Methods

### Derivation of analysis results

In order to derive the analysis expression of the probability distribution and noise, we transform the chemical master equation into a confluence hypergeometric equation [51,53], using the probability-generating function.

### Stochastic simulations

Stochastic simulations of our model studied here have been carried out using the Gillespie algorithm [76].

## Additional files

Additional file 1:
**Effects of promoter leakage on expression dynamics in more complex situations. Figure S1.** The effect of promoter leakage on expression dynamics in a gene auto-repressing model that assumes the dissociation of transcription factors from genomic binding site is a slow process. **Figure S2.** The effect of promoter leakage on expression dynamics in a gene auto-enhancing model that assumes the dissociation of transcription factors from genomic binding site is a slow process. **Figure S3.** Effects of promoter leakage on distribution and noise in a full two-state gene model. **Figure S4.** The effect of promoter leakage on burst dynamics in the case that amount of the gene product is fixed. Additional file 2:
**Theoretical derivations of probability and noise.**

## Abbreviations

BS: Burst size
BF: Burst frequency
CME: Chemical master equation
