# Supplementary material for: Effects of promoter leakage on dynamics of gene expression
Source: BMC Syst Biol. 2015 Mar 21;9:16. doi: 10.1186/s12918-015-0157-z (PMC4384279; doi:10.1186/s12918-015-0157-z)
Supplement: Additional file 1: — Effects of promoter leakage on expression dynamics in more complex situations. Figure S1. The effect of promoter leakage on expression dynamics in a gene auto-repressing model that assumes the dissociation of transcription factors from genomic binding site is a slow process. Figure S2. The effect of promoter leakage on expression dynamics in a gene auto-enhancing model that assumes the dissociation of transcription factors from genomic binding site is a slow process. Figure S3. Effects of promoter leakage on distribution and noise in a full two-state gene model. Figure S4. The effect of promoter leakage on burst dynamics in the case that amount of the gene product is fixed. [file 12918_2015_157_MOESM1_ESM.docx]

**Additional file 1:Effects of promoter leakage on expression dynamics in more complex situations**

In this additional file, we present numerical results on how promoter leakage affects the distribution and noise of protein in more complex situations. The results are shown in

**Figure S1:** The effect of promoter leakage on expression dynamics in a gene auto-repressing model that assumes the dissociation of transcription factors from genomic binding site is a slow process.

**Figure S2:** The effect of promoter leakage on expression dynamics in a gene auto-enhancing model that assumes the dissociation of transcription factors from genomic binding site is a slow process.

**Figure S3:** Effects of promoter leakage on distribution and noise in a full two-state gene model.

**Figure S4:** The effect of promoter leakage on burst dynamics in the case that amount of the gene product is fixed.

**Figure S1**

**Effects of promoter leakage on distribution/noise in the case of self repression.** (A) **S**chematic diagram for the gene model, where gene is assumed to have one ON state with high efficiency and one OFF state with very low efficiency (termed as promoter leakage), and the dissociation of a transcription factor from genomic binding site is assumed as a slow process. The biochemical reactions for the full system take the form: $D_{0}\underset{\to}{\gamma_{1}}D_{1}, D_{1}\underset{\to}{\gamma_{0}}D_{0},$ $D_{1}+P\underset{\to}{f_{1}}D_{0}, D_{0}\underset{\to}{f_{2}}D_{1}+P, D_{1}\underset{\to}{\lambda_{1}}D_{1}+P, D_{0}\underset{\to}{\lambda_{0}}D_{0}+P, P\underset{\to}{\delta}\phi$, where $\lambda_{1}\gg\lambda_{0}$. (B) The gene product probability distribution from only one peak closed to the origin gradually becomes another peak away from the origin with increasing the leakage rate. Other parameters are set as $\gamma_{0}=0.1,\gamma_{1}=0.1,f_{1}=0.3,f_{2}=0.1,\lambda_{1}=0,\delta=1$. (C) Increasing the leakage rate always reduces the expression noise, where the parameter values are the same as those in (B).

**Figure S2**

**Effects of promoter leakage on distribution/noise in the case of self activation. (A) S**chematic diagram for the gene model, where gene is assumed to have one ON state with high efficiency and one OFF state with very low efficiency (termed as promoter leakage), and the dissociation of a transcription factor from genomic binding site is assumed as a slow process. The biochemical reactions for the full system take the form: $D_{0}\underset{\to}{\gamma_{1}}D_{1}, D_{1}\underset{\to}{\gamma_{0}}D_{0},$ $D_{1}+P\underset{\to}{f_{1}}D_{0}, D_{0}\underset{\to}{f_{2}}D_{1}+P, {D_{1}\underset{\to}{\lambda_{1}}D_{1}+P, D}_{0}\underset{\to}{\lambda_{0}}D_{0}+P, P\underset{\to}{\delta}\phi$, where $\lambda_{1}\ll\lambda_{0}$. (B) The gene product probability distribution changes from bimodality to unimodality with the increase of the leakage rate. Some parameter values are set as $\gamma_{0}=0.1,\gamma_{1}=0.1,f_{1}=0.1,f_{2}=0.3,\lambda_{0}=40,\delta=1$. (C) Increasing the leakage rate always reduce the expression noise, where the parameter values are the same as those in (B).

**Figure S3**

**Effects of promoter leakage on distribution/noise in a** **full two-state gene model.** (A) **S**chematic diagram for the gene model, where it is assumed that the promoter transitions between inactive and active states and there are two stochastic variables: one for mRNAs and the other for proteins. (B) The protein probability distribution changes from bimodality to unimodality with the increase of the leakage rate. Other parameters are set as $\gamma_{0}=0.1,\gamma_{1}=0.2,\lambda_{1}=30,\delta_{1}=10, \kappa=40,\delta_{2}=2$. (C) Increasing the leakage rate always reduces the noise in protein, where the parameter values are the same as those used in (B).

**Figure S4**

**Effects of promoter leakage on burst dynamics in the case that the mean level is fixed.** (A and B) negative feedback$(\lambda_{1}\gg\lambda_{0}$): promoter leakage ($\lambda_{0}$) always reduces burst size (BS) regardless of ways to keep the average level fixed, but burst frequency (BF) varies with compensating changes in other parameters. It is found that the mean BF basically no change with increasing the leakage rate, decreasing in the case of changing the transition rate from OFF to ON ($\gamma_{1}$) but keeping all the other parameter values fixed. The line corresponds to the theoretical solution of BF$\left\langle BF \right\rangle=\gamma_{1}$, whereas the other signs (e.g. circles) represent simulating solution. If the leakage rate ($\lambda_{0}$) is 0, then the other parameters are set as $\gamma_{0}=1,\gamma_{1}=1,\lambda_{1}=40,f=0.1, \delta=1$. (C and D) positive feedback ($\lambda_{1}\ll\lambda_{0}$): if increasing the leakage rate ($\lambda_{1}$) corresponds to the change of transition rate from OFF to ON ($\gamma_{0}$) or positive feedback intensity ($f$), then the mean BS does not change but the mean BF decreases; if increasing the leakage rate ($\lambda_{1}$) corresponds to the change of transition rate from ON to OFF ($\gamma_{1}$) or maximum transcription rate ($\lambda_{0}$), then the mean BS decreases and the mean BF increases. The line corresponds to the theoretical solution of burst frequency $\left\langle BF \right\rangle=\lambda_{0}/\gamma_{1}$, and the other signs (e.g. circles) represent simulating solution. If the leakage rate ($\lambda_{1}$) is 0, then the other parameters are set as $\gamma_{0}=1,\gamma_{1}=1,\lambda_{0}=40,f=0.1, \delta=1$.
