# Supplementary material for: Effects of promoter leakage on dynamics of gene expression
Source: BMC Syst Biol. 2015 Mar 21;9:16. doi: 10.1186/s12918-015-0157-z (PMC4384279; doi:10.1186/s12918-015-0157-z)
Supplement: Additional file 2: — Theoretical derivations of probability and noise. [file 12918_2015_157_MOESM2_ESM.docx]

**Additional file 2: Theoretical derivations of probability and noise**

Let $P_{0}(n,t)$ and $P_{1}(n,t)$represent the probability that the gen product has $n$ molecules at time $t$ when the gene is at $D_{0}$ and $D_{1}$ states, respectively. Then, the discrete CME for our gene model takes the form

$\frac{\partial}{\partial t}\left(\begin{matrix} P_{0}\left(n,t\right) \\ P_{1}\left(n,t\right) \end{matrix}\right)=\left(\begin{matrix} -\gamma_{1} & \gamma_{0}+fn \\ \gamma_{1} & -\gamma_{0}-fn \end{matrix}\right)\left(\begin{matrix} P_{0}\left(n,t\right) \\ P_{1}\left(n,t\right) \end{matrix}\right)+\left(\begin{matrix} \left[\lambda_{0}\left(E^{-1}-I\right)+d\left(E-I\right)\right]P_{0}\left(n,t\right) \\ \left[\lambda_{1}\left(E^{-1}-I\right)+d\left(E-I\right)\right]P_{1}\left(n,t\right) \end{matrix}\right)$ (S1)

where $E$ is the step operator and $I$ is the identity operator.

Next, we focus on finding the steady-state solution of the CME (S1). The basic idea is first to introduce probability-generating functions, and then to solve a coupled set of ordinary differential equations with respect to these functions. The overall procedure for finding the stationary distribution is technical.

Note that the transformation$G_{i}\left(z\right)=\sum_{n=0}^{\infty} P_{i}\left(n\right)z^{n}$become the discrete-variable function $P_{i}(n,t)$ into the continuous-variable function$G_{i}\left(z\right)$, where $i=0, 1$. Correspondingly, Eq. (S1) is transformed into the following ordinary differential equation group:

$\left(\begin{matrix} z-1 & -f z \\ 0 & z-1+f z \end{matrix}\right)\frac{d}{dz}\left(\begin{matrix} G_{0} \\ G_{1} \end{matrix}\right)-\left(\begin{matrix} \lambda_{0}\left(z-1\right)-\gamma_{1} & \gamma_{0} \\ \gamma_{1} & \lambda_{1}\left(z-1\right)-\gamma_{0} \end{matrix}\right)\left(\begin{matrix} G_{0} \\ G_{1} \end{matrix}\right)=\left(\begin{matrix} 0 \\ 0 \end{matrix}\right)$ (S2)

where all the parameters are normalized by the degradation rate $d$, that is, $\gamma_{1}/d\to\gamma_{1}$,$\gamma_{0}/d\to\gamma_{0}$, $f/d\to f$, $\lambda_{0}/d\to\lambda_{0}$, $\lambda_{1}/d\to\lambda_{1}$. Adding two equations in Eq. (S2) yields the following relation between $G_{0}\left(z\right)$ and $G_{1}\left(z\right)$

$\lambda_{0}G_{0}-G'_{0}=-\left(\lambda_{1}G_{1}-G'_{1}\right)$ (S3)

Hereafter the derivative is with respect to$z$.

In spite of this relation at hand, solving Eq. (S2) is still not easy since its coefficient matrices are not constant. Here, we introduce a pair of transformations given by

$H_{0}\left(z\right)=e^{\lambda_{0}z}G_{0}(z)$, $H_{1}\left(z\right)=e^{\lambda_{1}z}G_{1}(z)$ (S4)

which transform the relation between $G_{0}\left(z\right)$ and $G_{1}\left(z\right)$ given by Eq. (S3) into that between $H_{0}\left(z\right)$ and $H_{1}\left(z\right)$

$e^{\lambda_{1}z}H'_{1}(z)=-e^{\lambda_{0}z}H'_{0}(z)$ (S5)

and Eq. (S2) into the following coupled equation group

$-\gamma_{1}e^{\lambda_{0}z}H_{0}(z)+\gamma_{0}e^{\lambda_{1}z}H_{1}(z)+fz\left[\lambda_{1}e^{\lambda_{1}z}H_{1}(z)+e^{\lambda_{1}z}H'_{1}(z)\right]-\left(z-1\right)e^{\lambda_{0}z}H'_{0}(z)=0$ (S6a)

$\gamma_{1}e^{\lambda_{0}z}H_{0}(z)-\gamma_{0}e^{\lambda_{1}z}H_{1}(z)-fz\left[\lambda_{1}e^{\lambda_{1}z}H_{1}(z)+e^{\lambda_{1}z}H'_{1}(z)\right]-\left(z-1\right)e^{\lambda_{1}z}H'_{1}(z)=0$ (S6b)

From this equation combined with Eq. (S3), it is not difficult to derive the following second-order differential equation of $H_{1}\left(z\right)$

$A\left(z\right)H_{1}^{' '}(z)+B\left(z\right)H_{1}^{'}(z)+C\left(z\right)H_{1}(z)=0$ (S7)

where the coefficients are

$A\left(z\right)=\left(f+1\right)z-1$ (S8a)

$B\left(z\right)=\left(\lambda f+\lambda+f \lambda_{1}\right)z+\gamma_{0}+\gamma_{1}+f+1-\lambda$ (S8b)

$C\left(z\right)=f \lambda\lambda_{1}z+\gamma_{0}\lambda+f \lambda_{1}$ (S8c)

$\lambda=\lambda_{1}-\lambda_{0}$ (S8d)

Now, we readily transform Eq. (S7) into a confluent hypergeometric equation. In fact, the function transformation $H_{1}(z)=e^{-\lambda z}f\left(\omega\right)$ with

$\omega=\left[\left(f+1\right)z-1\right]\frac{\lambda-f \lambda_{0}}{(f+1)^{2}}$ (S9)

yields the following standard confluent hypergeometric equation

$\omega f''(w)+\left(\beta-w\right)f'(w)-\alpha f(w)=0$ (S10)

which is our familiar, where a pair of system parameters $\alpha$ and $\beta$ are given by

$\alpha=1+\frac{\lambda\gamma_{1}}{R}$ and $\beta=1+\frac{\lambda+\gamma_{0}+\gamma_{1}}{f+1}-\frac{R}{\left(f+1\right)^{2}}$ with $R=\lambda-f \lambda_{0}$ (S11)

Note that Eq. (S10) admits two independent solutions: the one is the confluent hypergeometric function ${}_{1}F{}_{1}\left(\alpha, \beta; \omega\right)$ and the other is the Tricomi function $U\left(\alpha, \beta; \omega\right)$. However, the latter solution is infeasible since we require $P_{1}(n)\to0$ for $n\to\infty$ and that the mean of $n$ must be finite. Thus, we can use have ${}_{1}F{}_{1}\left(\alpha, \beta; \omega\right)$ to express the function $H_{1}\left(z\right)$ and further the function $G_{1}\left(z\right)$ using the relationship between variables. The result is

$G_{1}\left(z\right)=e^{\lambda_{1}z}H_{1}(z)=Ae^{\lambda_{0}z}{}_{1}F_{1}\left(\alpha, \beta; \omega\right)$ (S12)

where $A$ is a normalization constant and the relationship between $z$ and $\omega$ is determined by Eq. (S9). Using this analytical expression combined with the relationship between $H_{0}\left(z\right)$ and $H_{1}\left(z\right)$ given by Eq. (S5), we obtain the following first-order differential equation of $H_{0}\left(z\right)$

$H'_{0}(z)=A\left[\lambda{}_{1}F_{1}\left(\alpha, \beta; \omega\right)-\frac{\alpha}{\beta}(f+1)Q{}_{1}F_{1}\left(\alpha+1, \beta+1; \omega\right)\right]$ (S13)

which gives the following analytical expression for $H_{0}(z)$

$H_{0}\left(z\right)=A\left[g{}_{1}F_{1}\left(\alpha-1, \beta-1; \omega\right)-{}_{1}F_{1}\left(\alpha, \beta; \omega\right)\right]+C$ (S14)

with $Q=\frac{\lambda-f \lambda_{0}}{(f+1)^{2}}$ and $g=\frac{\lambda+\gamma_{1}+\gamma_{0}}{\gamma_{1}}-\frac{R}{\gamma_{1}\left(f+1\right)}$, where $C$ is a certain constant, which can be determined by the conservation condition of probability: $\sum_{n=0}^{\infty} \left[P_{0}(n)+P_{1}(n)\right]=1$ or equally $G_{0}\left(1\right)+G_{1}\left(1\right)=1$. As a result, we find $C=0$. Using the relationship between $H_{0}(z)$ and $G_{0}(z)$, we thus obtain the analytical expression of $G_{0}(z)$ as

$G_{0}\left(z\right)=Ae^{\lambda_{0}z}\left[g{}_{1}F_{1}\left(\alpha-1, \beta-1; \omega\right)-{}_{1}F_{1}\left(\alpha, \beta; \omega\right)\right]$ (S15)

which is the linear superposition of two confluent hypergeometric functions, where the relationship between $z$ and $\omega$ is determined by Eq. (S9), and

$A=e^{-\lambda_{0}}\left[g{}_{1}F_{1}\left(\alpha-1, \beta-1; f Q\right)\right]^{-1}$ (S16)

Using the relationship between the probability distributions: $P_{0}(n)$ and $P_{1}(n)$, and the generating functions: $G_{0}(z)$ and $G_{1}(z)$

$P_{0}(n)=\left.\frac{1}{n!}\frac{d^{n}}{dz^{n}}G_{0}(z)\right|_{z=0}$, $P_{1}(n)=\left.\frac{1}{n!}\frac{d^{n}}{dz^{n}}G_{1}(z)\right|_{z=0}$ (S17)

we finally obtain the analytical expression for the total probability distribution as

$P(n)=\frac{gA}{n!}\sum_{m=0}^{n} \left(\begin{matrix} n \\ m \end{matrix}\right)\lambda_{0}^{n-m}\left[(f+1)Q\right]^{m}\frac{(\alpha-1)_{m}}{(\beta-1)_{m}}{}_{1}F_{1}\left(\alpha+m-1, \beta+m-1; -Q\right)$ (S18)

which is the linear superposition of confluent hypergeometric functions. Here $\left(\begin{matrix} n \\ m \end{matrix}\right)$ represents the combination number of choosing $m$ molecule from $n$ molecules, and $\left(c\right)_{n}$ is the Pochhammer symbol and is defined as$\left(c\right)_{n}=\Gamma\left(c+n\right)/\Gamma\left(c\right)$. In principle, this analytical distribution gives all the stochastic information about the underlying gene model.

In particular, the analytical distribution given by Eq. (S18) includes all the previously-obtained distributions as its special case. For example, suppose that the gene is expressed in a constitutive manner. Then, the corresponding gene model corresponds to our case with $\gamma_{1}=\gamma_{0}=0$, $f=0$ and $\lambda_{0}=0$. In this case, we have$Q=\lambda_{1}$, $R=\lambda_{1}$, $g=1$, $\alpha=1$, $\beta=1$ and $A=1$. Therefore, $P\left(n\right)$ given by Eq. (S18) is reduced to

$P(n)=\frac{1}{n!}\frac{(0)_{n}}{(0)_{n}}\lambda_{1}^{n}{}_{1}F_{1}\left(n, n; -\lambda_{1}\right)=\frac{\lambda_{1}^{n}}{n!}e^{-\lambda_{1}}$ (S19)

which is a Poisson distribution with the characteristic parameter $\lambda_{1}$, in accord to the previous-obtained result [1] by noting the rescaled parameters. Suppose that the gene is expressed in a bursty manner. Then, the corresponding gene model corresponds to our case with $f=0$, $\lambda_{0}=0$. In this case, we have$Q=\lambda_{1}$,$R=\lambda_{1}$, $g=\frac{\gamma_{1}+\gamma_{0}}{\gamma_{1}}$, $\alpha=1+\gamma_{1}$, $\beta=1+\gamma_{0}+\gamma_{1}$, and $A=\frac{\gamma_{1}}{\gamma_{1}+\gamma_{0}}e^{-\lambda_{0}}$. Therefore, $P\left(n\right)$ given by Eq. (S18) is reduced to

$P(n)=e^{-\frac{\lambda_{0}}{d}}\frac{\left(\lambda_{1}/d\right)^{n}}{n!}\frac{\left(\gamma_{1}/d\right)_{n}}{\left(\gamma_{0}/d+\gamma_{1}/d\right)_{n}}{}_{1}F_{1}\left(n+\frac{\gamma_{1}}{d}, n+\frac{\gamma_{0}}{d}+\frac{\gamma_{1}}{d}; -\frac{\lambda_{1}}{d}\right)$ (S20)

by recovering the original parameters, which reproduces the previous distribution [2]. These two examples indicate that the analytical distribution given by Eq. (S18) is without doubt correct.

The more useful is that we can give the analytical formula for calculating the noise intensity for the gene product that is defined as the ratio of the variance over the mean. In fact, note that the mean and the variance are calculated according to

$\left\langle n\right\rangle=G'(1)$, $\sigma_{n}^{2}=G''(1)+G'(1)-\left[G'(1)\right]^{2}$ (S21)

where$G(z)=G_{0}(z)+G_{1}(z)$ is the total generating function. Two functions $G_{0}\left(z\right)$ and $G_{1}\left(z\right)$ is analytically given as seen above, so is the function$G\left(z\right)$. By calculation, we find

$G'(1)=Ae^{\lambda_{0}}\left[g\lambda_{0}{}_{1}F_{1}\left(\alpha-1, \beta-1, f Q\right)+\lambda{}_{1}F_{1}\left(\alpha, \beta, f Q\right)\right]$ (S22)

$\begin{matrix} G''(1)=Ae^{\lambda_{0}}\left[g\lambda_{0}^{2}{}_{1}F_{1}\left(\alpha-1, \beta-1, f Q\right)+2\lambda\lambda_{0}{}_{1}F_{1}\left(\alpha, \beta, f Q\right)\right. \\ +\frac{\lambda\left(\lambda\gamma_{1}+R\right)}{\lambda+\gamma_{1}+\gamma_{0}+f+1-\frac{R}{f+1}}{}_{1}F_{1}\left(\alpha+1, \beta+1, f Q\right)] \end{matrix}$ (S23)

where$A$, $g$, $Q$, $R$, $\alpha$ and $\beta$ are compounded parameters, whose expressions have been given in the above. Thus, the square of the noise intensity is calculated according to the formula

$\eta_{n}^{2}=\frac{\sigma_{n}^{2}}{\left\langle n\right\rangle{}^{2}}=\frac{G''(1)+G'(1)-\left[G'(1)\right]^{2}}{\left[G'(1)\right]^{2}}$ (S24)

Using this analytical expression, we can reproduce previous results on the noise in the gene product [3，4]. For example, if there are no feedback (i.e., $f=0$) and no promoter leakage (e.g., $\lambda_{0}=0$), then the mean, the variance and the square of the noise intensity are given respectively by

$\left\langle n\right\rangle=\frac{\gamma_{1}\lambda_{1}}{\gamma_{1}+\gamma_{0}}$,$\sigma_{n}^{2}=\frac{\lambda_{1}^{2}\gamma_{0}\gamma_{1}+\lambda_{1}\gamma_{1}\left(\gamma_{0}+\gamma_{1}\right)\left(1+\gamma_{0}+\gamma_{1}\right)}{\left(\gamma_{0}+\gamma_{1}\right)^{2}\left(1+\gamma_{0}+\gamma_{1}\right)}$, $\eta_{n}^{2}=\frac{1}{\gamma_{1}}\frac{\gamma_{0}}{1+\gamma_{0}+\gamma_{1}}+\frac{\gamma_{0}+\gamma_{1}}{\gamma_{1}\lambda_{1}}$(S25)

which is a known result [3]. In particular, we can deduce the dependence of the expression noise on feedback strength, with the qualitative result being in accord with the previous one [5, 6]. There are many results for simplified versions of our model.

**Supplementary references**

1. Kampen V, Godfried Nicolaas: ***Stochastic processes in physics and chemistry*.** Vol. 2. Elsevier, 2007.
2. Shahrezaei V, Swain PS: **Analytical distributions for stochastic gene expression.** Proc Natl Acad Sci USA. 2008, **105(45):** 17256-17261.
3. Kepler TB, Elston TC: **Stochasticity in transcriptional regulation: origins, consequences, and mathematical representations.** Biophys J 2001, **81(6):** 3116-3136.
4. Zhang JJ, Nie Q, He M, Zhou TS: **An effective method for computing the noise in biochemical networks.** J Chem Phys 2013, **138(8):** 084106.
5. Paulsson J: **Summing up the noise in gene networks.** Nature 2004, **427(6973):** 415-418.
6. Marquez-Lago TT, Stelling J: **Counter-intuitive stochastic behavior of simple gene circuits with negative feedback.** Biophys J 2010, **98(9):** 1742-1750.
